# Subacute sclerosing pan encephalitis with HIV: two rare case reports

**DOI:** 10.1186/1471-2334-14-S3-O25

**Published:** 2014-05-27

**Authors:** N Hazra, R Mani, A Desai, S Sinha, M Netravathi, AB Taly, P Satishchandra, V Ravi

**Affiliations:** 1 Dept of Neurovirology, NIMHANS, Bangalore, India; 2 Dept of Neurology, NIMHANS Bangalore, India; 3 Director/Vice Chancellor, NIMHANS Bangalore, India

## Background

Subacute Sclerosing Pan Encephalitis (SSPE) is a rare, chronic neurological disease of children and adolescents resulting from persistent measles virus infection of neurons. Not only is the occurrence of SSPE in HIV infection rare, also the natural history has not yet been elucidated.

## Case Reports

Two children, a 10 year old boy and an 11 year old girl, both HIV-1 seropositive following perinatally acquired infection, presented with strikingly different clinical symptoms to Neurology OPD, NIMHANS. The boy presented with deterioration in scholastic performance over a four month duration with generalized tonic clonic seizures of 1 month duration. Vision was normal. The girl was found to have evidence of bilateral blindness, altered gait with dysarthric speech and severe truncal ataxia.

Both children were unvaccinated for measles and gave a definitive history of Measles at the age of 8 months and 6 years. The latent period between Measles infection and SSPE was 9 and 5 years respectively. Clinically both were staged at Jabbour Stage II and Gascon stage III. The CD4 counts were 554/mm^3^ and 130//mm^3^ respectively. In both patients, progressive cognitive decline, reduction in scholastic performance, regression of milestones and inability to walk with loss of self care occurred in 12 and 4 weeks respectively following onset.

## Conclusion

Presence of HIV resulted in a fulminant course with premature manifestation of myoclonic jerks and rapid-onset cognitive decline as well as rapid progressive deterioration of clinical staging in both patients.This report of two rare cases aims to stress the importance of awareness of the possible association of SSPE with HIV, especially with the increased life expectancy that is seen in HIV post HAART and the need to institute a global program to eliminate measles

